# Thermal Liquid Biopsy (TLB) of Blood Plasma as a Potential Tool to Help in the Early Diagnosis of Multiple Sclerosis

**DOI:** 10.3390/jpm11040295

**Published:** 2021-04-13

**Authors:** Ferdinanda Annesi, Sonia Hermoso-Durán, Bruno Rizzuti, Rosalinda Bruno, Domenico Pirritano, Alfredo Petrone, Francesco Del Giudice, Jorge Ojeda, Sonia Vega, Oscar Sanchez-Gracia, Adrian Velazquez-Campoy, Olga Abian, Rita Guzzi

**Affiliations:** 1 CNR-NANOTEC, Licryl-UOS Cosenza and CEMIF.Cal, Department of Physics, University of Calabria, 87036 Rende, Italy; ferdinanda.annesi@cnr.it (F.A.); bruno.rizzuti@cnr.it (B.R.); 2 Institute of Biocomputation and Physics of Complex Systems (BIFI), Joint Units IQFR-CSIC-BIFI, and GBsC-CSIC-BIFI, Universidad de Zaragoza, 50018 Zaragoza, Spain; shermosod@gmail.com (S.H.-D.); svega@bifi.es (S.V.); adrianvc@unizar.es (A.V.-C.); 3 Instituto de Investigación Sanitaria Aragón (IIS Aragón), 50009 Zaragoza, Spain; 4 Department of Pharmacy, Health and Nutritional Sciences, University of Calabria, 87036 Rende, Italy; rosalinda.bruno@unical.it; 5 Neurological and Stroke Unit, Multiple Sclerosis Clinic, Annunziata Hospital, 87100 Cosenza, Italy; pirritanodomenico@gmail.com (D.P.); alpetrone@gmail.com (A.P.); fra.delgiu@libero.it (F.D.G.); 6 Department of Statistical Methods, Universidad de Zaragoza, 50009 Zaragoza, Spain; jojeda@unizar.es; 7 SOTER BioAnalytics, Enrique Val, 50011 Zaragoza, Spain; oscar.sanchez.gracia@gmail.com; 8 Departamento de Bioquímica y Biología Molecular y Celular, Universidad de Zaragoza, 50009 Zaragoza, Spain; 9 Centro de Investigación Biomédica en Red en el Área Temática de Enfermedades Hepáticas y Digestivas (CIBERehd), 28029 Madrid, Spain; 10 Fundación ARAID, Gobierno de Aragón, 50009 Zaragoza, Spain; 11 Instituto Aragonés de Ciencias de la Salud (IACS), 50009 Zaragoza, Spain; 12 Department of Physics, Molecular Biophysics Laboratory, University of Calabria, 87036 Rende, Italy

**Keywords:** multiple sclerosis, thermal liquid biopsy, plasma proteome profile, differential scanning calorimetry, multivariate analysis

## Abstract

Background: Multiple sclerosis (MS) is frequently characterized by a variety of clinical signs, often exhibiting little specificity. The diagnosis requires a combination of medical observations and instrumental tests, and any support for its objective assessment is helpful. Objective: Herein, we describe the application of thermal liquid biopsy (TLB) of blood plasma samples, a methodology for predicting the occurrence of MS with a noninvasive, quick blood test. Methods: TLB allows one to define an index (TLB score), which provides information about overall real-time alterations in plasma proteome that may be indicative of MS. Results: This pilot study, based on 85 subjects (45 MS patients and 40 controls), showed good performance indexes (sensitivity and specificity both around 70%). The diagnostic methods better discriminate between early stage and low-burden MS patients, and it is not influenced by gender, age, or assumption of therapeutic drugs. TLB is more accurate for patients having low disability level (≤ 3.0, measured by the expanded disability status scale, EDSS) and a relapsing–remitting diagnosis. Conclusion: Our results suggest that TLB can be applied to MS, especially in an initial phase of the disease when diagnosis is difficult and yet more important (in such cases, accuracy of prediction is close to 80%), as well as in personalized patient periodic monitoring. The next step will be determining its utility in differentiating between MS and other disorders, in particular in inflammatory diseases.

## 1. Introduction

Multiple sclerosis (MS) is the most common neurodegenerative disease of the central nervous system (CNS) in young adults, ultimately leading to long term disability [1]. It is characterized by a chronic inflammatory status causing demyelination of neurons and axonal loss. MS incidence is increasing worldwide (more than 2.8 million people are believed to be currently affected), prevailing in more-developed countries, high-income people, and with women showing a greater propensity (female to male ratio is 3 to 1) to develop the disease [2,3]. On a clinical basis, a variety of neurological signs and symptoms may occur, including sensory disturbances, motor weakness, visual complaints, incoordination, fatigue, sphincteric, and sexual dysfunction [4].

Due to the variety of potential indicators, MS diagnosis is very challenging. Its identification is primarily clinical and relies on the documentation of symptoms and signs attributable to white matter lesions, along with the exclusion of other conditions that may mimic MS. There is no single pathognomonic clinical feature or diagnostic test of MS, and its detection is based on the integration of clinical, imaging, and laboratory findings. Diagnosis can be supported by cerebrospinal fluid (CSF) analysis, whereas blood tests are commonly used only to rule out other diseases. In such a complex picture, physical disability is commonly quantified by the expanded disability status scale (EDSS), which integrates neurological findings concerning eight functional systems and mainly relies on the assessment of the patient walking ability. The EDSS score ranges from 0 (completely normal neurological examination) to 10 (death due to MS), with values >7 indicating a very compromised clinical picture.

The rate of MS misdiagnosis is about 10% and represents an issue in the clinical practice [5]. The risk of misdiagnosis is particularly present in the early stages of the disease, when symptoms are often mild, generic, or common to other different disorders. Moreover, there is a high interest in attaining a timely diagnosis to allow the patient to benefit of an early treatment, which may further increase misdiagnosis risks based on such mild symptoms. In order to make a diagnosis of MS, the 2017 McDonald criteria stress the need for no better explanation to account for the variety of symptoms observed [6]. In this framework, it would be very desirable to combine current diagnostic methods with innovative complementary biomarkers able to identify new indicative parameters, especially in the early stage of the disease, when the current methods may not fully capture the pathological signs.

In the latest years, a new area of interest is the search of novel biomarkers to help diagnosis and to monitor ongoing disease activity. CSF represents the ideal source of biomarkers, but lumbar puncture is a high-risk relatively invasive procedure and unpleasant for the patients; therefore, it is not an optimal tool. In contrast, easily collected blood samples may reflect the status of both peripheral immune system and, indirectly, of CNS functioning mechanisms [7,8,9,10]. In fact, about 0.5 L of CSF is adsorbed into the blood every day, suggesting that plasma may be a source of disease biomarkers originating from CSF [11].

To detect and quantify biological materials ranging from proteins to disease-specific biomarkers, high-sensitivity biophysical techniques are available [12,13,14,15]. These methods include differential scanning calorimetry (DSC), a thermoanalytical technique widely applied in life science to measure the thermal denaturation profiles of biomolecules and their interaction with various metabolites [16,17,18,19,20]. More recently, this method is expanding in the biomedical area to monitor the thermal behavior of complex biological fluids, such as plasma, CSF, or other extracts obtained from homogenates tissue [21,22,23,24,25]. The plasma thermal profile (thermogram) reflects the thermal denaturation of the major plasma proteins, revealing changes in composition and the presence of post-translational modifications and/or interacting metabolites in a global way. Interestingly, comparison of the thermal profiles of biological samples among healthy and pathologic individuals has revealed distinctive alteration, contributing to validate DSC as a complementary noninvasive tool for the diagnosis and discrimination of several autoimmune diseases and malignancies, including gastric adenocarcinoma [24], lupus [26], glioblastoma [25], lung cancer [27], and melanoma [28].

A further and more ambitious step in this direction is the clinical implementation of the thermal liquid biopsy (TLB), which combines the extraction of physical parameters derived from the experimental DSC thermograms of blood samples with a multiparametric mathematical analysis [27,28]. The aim of TLB is to obtain a score value representing a single diagnostic indicator able to predict the occurrence of a disease, to be easily compared with a control reference value. The TLB has already been applied to study lung cancer, providing a prediction score that strongly correlates with the presence of disease, with high accuracy, sensitivity, and specificity [27].

In the present work, we apply TLB to discriminate MS patients from healthy individuals by analyzing their plasma thermograms. The plasma thermal profile (thermogram) reflects the thermal denaturation of the major plasma proteins, revealing changes in composition, and the presence of post-translational modifications and/or interacting metabolites in a global way. The results show clear statistical differences between the two groups of individuals. The TLB score is able to capture, with a good reliability, thermal profile alterations of plasma of MS patients. Thus, we suggest that TLB can help to improve MS diagnostic and monitoring, in combination with other tests, and constitutes a noninvasive, low-risk, quick diagnostic tool.

## 2. Materials and Methods

### 2.1. Subjects

MS patients, with a confirmed MS diagnosis according to the revised McDonald criteria [6,29], were recruited in the MS Center of the Annunziata Hospital (Cosenza, Italy). Exclusion criteria were: concomitant autoimmune disorders other than MS, pregnancy, and a high degree of cognitive decline preventing the expression of an informed consent. The collected clinical and personal data of the patients included gender, age, disease duration, disease severity expressed in terms of EDSS, and current pharmacological treatment.

**Figure 1:**
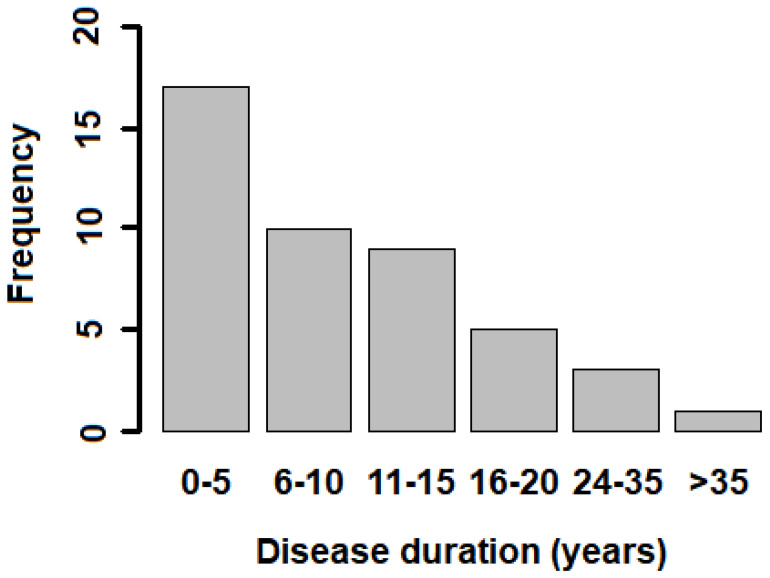
Distribution of disease onset time for MS patients.

The MS group consisted of 45 patients (31 females and 14 males), in the range of age 22–69 years (average 42.7 years) (Table 1). Most of the patients had a relapsing–remitting clinical form of MS (RRMS), and few of them were in the secondary progressive (SPMS) phase (Table 1). The EDSS values in the MS cohort ranged from 0.5 to 7.0 (0.5–3.0: mild disability; 3.5‒7.0: moderate/severe disability). Most of the patients (32 individuals) had EDSS values in the 0.5–3.0 range, whereas 13 of them belonged to the 3.5–7.0 range (Table 1). Within the MS group, the median age of patients in the EDSS = 3.5–7.0 group (49.85 (8.59)) is significantly higher (*p* = 0.003, *T*-test) than the median age of patients in the EDSS = 0.5–3.0 group (39.75 (11.04)). The time from the onset of the disease was very variable, ranging from 1 to 47 years (Table 1 and Figure 1). All the patients, with the exception of seven of them, were treated with immunomodulatory or suppressive therapy (Table 1).

The HC group consisted of 40 individuals, including 21 females and 19 males (Table 1). They were recruited at the same Annunziata Hospital (21 subjects) or among blood donors in the Centro Sanitario of the University of Calabria (19 subjects). According to the Fisher test (*p* = 0.181), there were no statistical difference in the proportion of males and females in the HC group and in the MS group. The age range was 24–60 years with a single outlier of 71 years (average age: 37.3 years), and all of them showed no evidence of inflammatory and neurological diseases. There were statistically significant differences (*p* = 0.020, Wilcox test) between the median age of the HC group (35.00 [29.00;42.25]) and the median age of the MS group (45.00 [33.00;50.00]), being higher in the MS group.

All MS and HC subjects had the same ethnic origin (Calabria, Italy). They were fully informed about the purpose of the study and gave a written consent. The study was approved by the Ethics Committee of the Northern Area of the Calabria Region (protocol n. 50 of February 14, 2017).

### 2.2. Blood Sample Processing

Samples (3 mL) of peripheral venous blood of the subjects were collected in EDTA tubes, and plasma was separated by centrifugation at 1500 rpm for 15 min. Processed plasma was dispensed in 0.1 mL aliquots and stored at −20 °C until use. Total protein concentration was measured before freezing, by using the Biuret method [30].

### 2.3. Sample Preparation and DSC Measurements

After thawing, plasma samples were diluted 1:20 (*v*/*v*) with Dulbecco phosphate buffer saline (DPBS) solution (Sigma Aldrich, St. Louis, MI, USA), 10 mM at pH 7.4, and properly degassed before being loaded into the cell. Thermograms were registered with a high-sensitivity VP-DSC microcalorimeter (MicroCal, Northampton, MA, USA) at a scan rate of 1 °C/min between 10 and 100 °C, after 20 min of equilibration time at the starting temperature. Solvent–solvent baselines acquisition followed the same experimental conditions. Samples were given a code, and lab technicians (performing the thermal denaturation experiments and analyzing the experimental data) did not know the nature of each sample, ensuring that this study was conducted in a blind manner.

### 2.4. Thermogram Analysis and Deconvolution

Correction and processing of the raw data was performed using Origin software (OriginLab, Northampton, MA) as previously described [24]. In brief, a multiparametric procedure was applied, based on a deconvolution analysis of each thermogram with six individual components. The mathematical model for each of the individual transitions is the logistic peak function:

(1) $$C_{p}\left(T\right)=C_{p,0}+\overset{i=6}{\underset{i=1}{\sum }}\frac{4A_{i}exp\left(\frac{-\left(T-T_{c,i}\right)}{w_{i}}\right)}{{\left(1+exp\left(\frac{-\left(T-T_{c,i}\right)}{w_{i}}\right)\right)}^{2}}$$

where each peak is characterized by three parameters: the height, *A_i_*, the center temperature, *T_c,i_*, and the width, $w_{i}$. Moreover, *C*_*p*,0_ is an adjustable parameter to offset the baseline correction. The deconvolution analysis provides parameters describing phenomenological physical features for each experimental thermogram. Observed thermogram alterations due to up- or down-regulation of proteins/components in plasma or due to interactions among proteins and metabolites would be reflected in such parameters. These eighteen parameters represent the primary set of the transition parameters for the subsequent mathematical processing.

### 2.5. Multiparametric Data Analysis

The 18 primary parameters obtained directly from the thermogram deconvolution were combined to define a new final set of 14 parameters, {*p_k_*}, more convenient for extracting and summarizing the essential geometric thermogram features to be used for calculating the TLB score and comparing thermograms, as defined in [24]. The final parameters obtained from the calorimetric curves were defined as follows:

The average temperature, *T_av_*, describes the average temperature of the thermogram *C_p_*(*T*) when considered as a density distribution function:

(2) $$T_{av}=\frac{\sum_{j}C_{p}\left(T_{j}\right)T_{j}}{\sum_{j}C_{p}\left(T_{j}\right)}$$

with *j* running over the entire range of the experimental points in the thermogram.

The skewness, *G*_1_, describes the asymmetry of the thermogram:

(3) $$\begin{array}{c} G_{1}=\frac{m_{3}}{m_{2}^{3/2}}\\ m_{k}=\frac{\sum_{j}C_{p}\left(T_{j}\right){\left(T_{j}-T_{av}\right)}^{k}}{\sum_{j}C_{p}\left(T_{j}\right)} \end{array}$$

The normalized area under the curve, *AUC_ni_*, provides an *A_i_*-normalized area under the thermogram:

(4) $$AUC_{ni}=\frac{\sum_{j}C_{p}\left(T_{j}\right)}{A_{i}}$$

The normalized area of the height polygon, *AP_ni_*, provides the *A_i_*-normalized area of the irregular hexagonal plot constructed with the heights of the six individual components:

(5) $$AP_{ni}=\overset{s=6}{\underset{s=1}{\sum }}\frac{\sqrt{3}}{4}\frac{A_{s}A_{s+1}}{A_{i}^{2}}$$

In addition, finally, the normalized distance value, *Dv_i_*, provides the Euclidean distance, using *T_av_*, *G*_1_, and *AP_ni_*, as Cartesian coordinates, from the center of the HC group ellipsoid (geometric point with coordinates equal to the average values for those parameters within the set of the healthy individuals):

(6) $$Dv_{i}=\sqrt{{\left(\frac{T_{av}-\bar{T_{av}}}{\bar{T_{av}}}\right)}^{2}+{\left(\frac{G_{1}-\bar{G_{1}}}{\bar{G_{1}}}\right)}^{2}+{\left(\frac{AP_{ni}-\bar{AP_{ni}}}{\bar{AP_{ni}}}\right)}^{2}}$$

### 2.6. Statistical Model

The final set of parameters {*p_k_*} obtained in the multiparametric data analysis were used to derive the *TLB* score by using a generalized linear model (GLM), which represents the probability, *P*, of an individual to show plasma alterations in the thermogram according to its plasma thermogram characterized by {*p_k_*}, which could be associated to disease:

(7) $$TLB\;score=P\left(alterations|\left\{p_{k}\right\}\right)=\frac{e^{\left(\mu \left(\left\{p_{k}\right\}\right)\right)}}{1+e^{\left(\mu \left(\left\{p_{k}\right\}\right)\right)}}$$

where *µ*({*p_k_*}) is a linear combination of the final parameters derived in the previous section (see Equations (2)–(6)).

(8) $$\mu \left(\left\{p_{k}\right\}\right)=a_{0}+\underset{k=1}{\sum }a_{k}p_{k}$$

The coefficients optimal *a_k_* are estimated by means of a maximum likelihood estimator (binomial GLM with logit link model). Because the purpose is predicting a binary variable (healthy vs. diseased), the binomial GLM becomes a suitable tool to estimate a logistic regression with the outcome being the probability plasma alteration (i.e., disease) according to a given TLB thermogram. Such a TLB score is the single value employed for classifying a given subject as healthy or diseased (diagnostic test). As any probability, the TLB score ranges between 0 and 1: the model classifies the subjects as having plasma alterations (diseased) for TLB score values >0.5 and lacking relevant plasma alterations (healthy) for values <0.5.

The performance of the diagnostic test was evaluated by calculating common performance indexes (sensitivity, specificity, positive predictive value, and negative predictive value) and the receiver operating characteristic (ROC) curve.

(9) $$\begin{array}{c} \begin{array}{c} Sensitivity=\frac{TP}{TP+FN}\\ Specificity=\frac{TN}{TN+FP} \end{array}\\ \begin{array}{c} PPV=\frac{TP}{TP+FP}\\ NPV=\frac{TN}{TN+FN} \end{array} \end{array}$$

where *TP*, *TN*, *FP*, and *FN* refer to true positives, true negatives, false positives, and false negatives, respectively.

Previously, we employed the TLB score for lung cancer patient classification, and three closely related predictive models were constructed using the complete set or partial multiparametric sets of parameters mentioned above: model 1 based on *T_av_*, *G*_1_, *AUC_ni_*, and *AP_ni_* (10 parameters); model 2 based on *Dv_i_* (4 parameters); and model 3 based on all the 14 parameters [27]. In this work, we have applied the same methodology to MS for designing a model useful for patient diagnosis and monitoring.

## 3. Results

### 3.1. Thermograms of Plasma Samples for Subjects from HC and MS Groups

**Figure 2:**
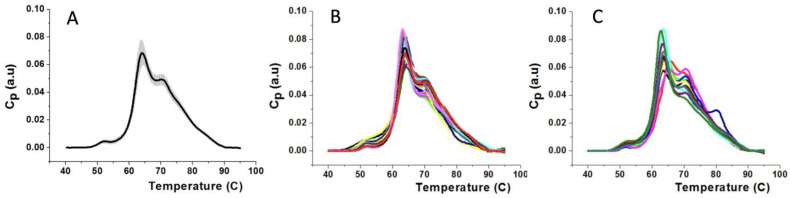
Thermogram comparison. (**A**) Average and standard deviation of the 40 thermograms from the HC group; (**B**) thermograms from 20 MS patients with EDSS ≤ 3.0; and (**C**) thermograms from 20 MS patients with EDSS > 3.5.

Thermograms reflecting the thermal behavior of the plasma proteins against thermal denaturation were acquired for all the subjects. Figure 2 shows some representative examples. The thermograms from the 40 HC individuals are very similar, providing a robust reference group for identifying differential features in MS patients. Visual inspection of thermograms from patients with EDSS ≤ 3 and EDSS > 3.5 did not provide any hints about key specific features of MS at any stage. Apart from individual variability, profile patterns seemed to be quite similar at a first glance.

### 3.2. Analysis of Individual TLB-Derived Parameters

**Figure 3:**
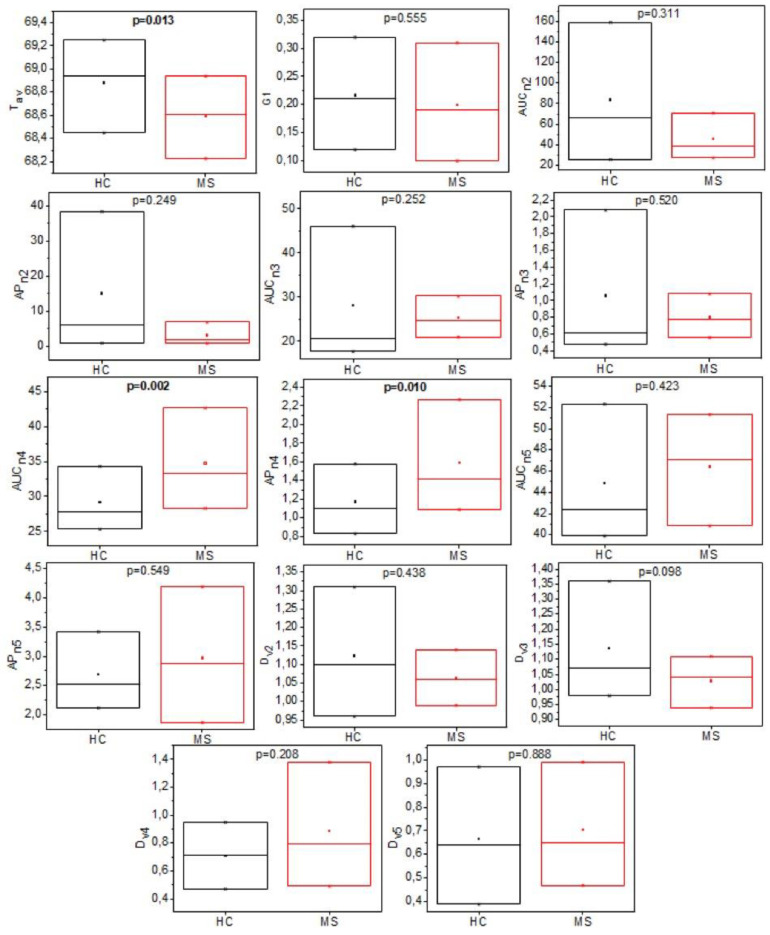
Box-plot for each individual parameter, derived from the deconvolution analysis of the thermograms, illustrating the distribution of their values in each group.

There were no statistically significant differences (*p* > 0.05, ANOVA test/Kruskal–Wallis test) of the mean/median of each TLB parameter as a function of group age (Table S1 included in the Supplementary Material). Within the MS group, there are no statistically significant differences (*p* > 0.05, ANOVA/Kruskal–Wallis test) of the mean/median of each TLB parameter as a function of the EDSS group, except for the parameter T_av_ (*p* = 0.011) (Table S2 included in the Supplementary Material). The thermograms for each individual were analyzed with the phenomenological six-component deconvolution curve, and the final set of fourteen TLB-associated parameters were calculated. As a preliminary statistical evaluation of the ability of each parameter to classify subjects into HC and MS groups, we determined the three boundaries (Q1, Q2, Q3) between the four quartiles for the distribution of each TLB-associated parameter within the two groups (Figure 3; Tables S3 and S4). According to Wilcoxon and t-Student tests, only *T_av_*, *AUC*_*n*4_ and *AP*_*n*4_ showed statistically significant differences between HC and MS groups (*p*-value < 0.05) (Table S4).

Subsequently, to further evaluate the predictive capabilities of each single TLB-associated parameter, we performed an individual ROC curve analysis. The idea behind such analysis is the identification of an optimal cut-off value for each parameter that might be employed for classifying subjects as either healthy or diseased (Table S5). The Youden method was employed [31]. It is evident that *AUC*_*n*3_, *AUC*_*n*4_, *AP*_*n*4_, *AUC*_*n*5_, *AP*_*n*5_, and *Dv*_5_ are the most successful individual parameters (high success rate, sensitivity, and specificity values) in correctly classifying the subjects.

### 3.3. TLB Score: A Classifying Predictor for MS

Because individual parameters are not able to discriminate efficiently between HC and MS groups, a multivariant logistic regression approach was applied employing the generalized linear model [27]. Three models were considered depending on the set of TLB-associated parameters included in this analysis (Supplementary Material, Table S6). Each model provided a TLB score for classifying subjects as healthy or diseased (<0.5 or >0.5, respectively). Model 3 (including all fourteen TLB-associated parameters) performed better compared to the other two models (Table 2 and Table S7), with a 68.24% success rate in correctly classifying individuals, 32.50% false positive rate (i.e., classifying a healthy subject as diseased), and 31.11% false negatives (i.e., classifying a diseased subject as healthy). No single parameter exhibited a statistical significance in the discrimination between HC and MS groups (Table S6); the combination of all parameters provided a TLB score with the highest ability to distinguish between HC and MS groups.

**Figure 4:**
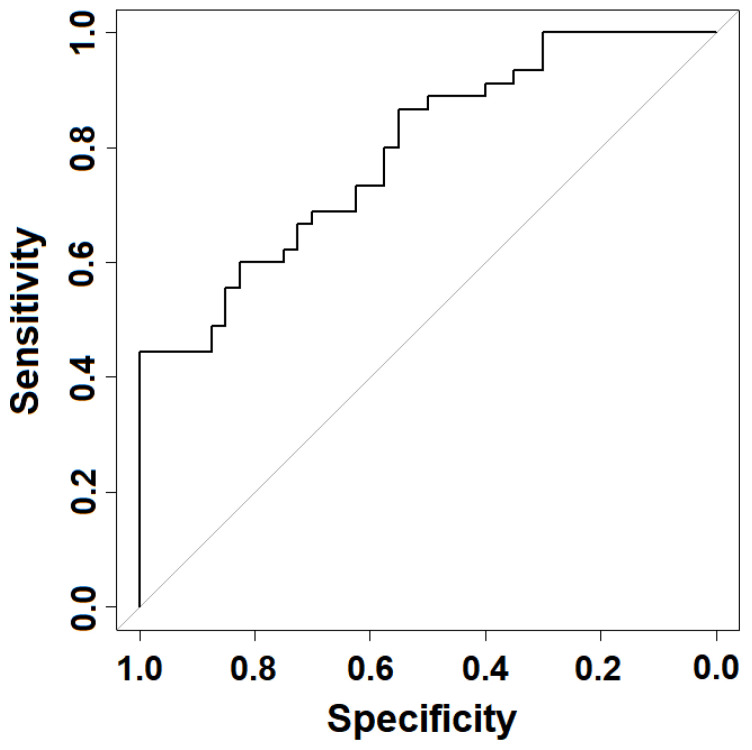
Receiver operating characteristic (ROC) curve illustrating the statistical performance of model 3 for calculating the thermal liquid biopsy (TLB) score.

The ability to discriminate between the two groups of subjects also reflected in the global ROC curve (Figure 4). The area under such curve was 0.79, indicating good accuracy of the methodology in terms of predictive power.

**Figure 5:**
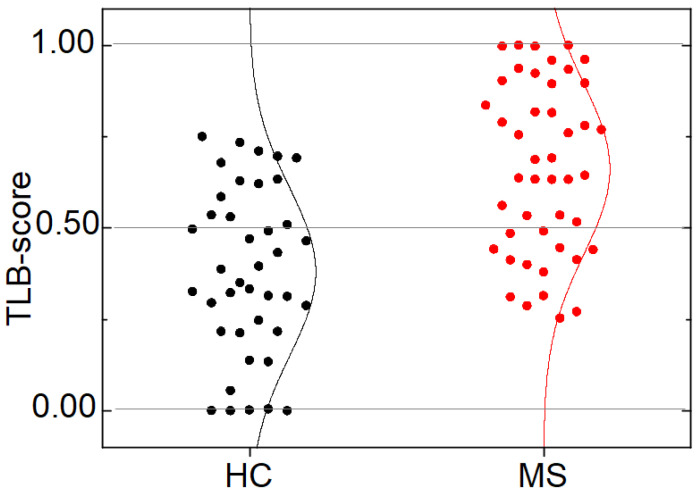
Distribution of the TLB score within healthy individuals (HC group) and multiple sclerosis patients (MS group). The lines represent an equivalent Gaussian distribution. The TLB score threshold for discriminating between an unaltered and altered thermal liquid biopsy (TLB) thermogram is 0.5. There are 13 HC subjects with TLB score > 0.5 (32.50% false positive rate) and 14 MS patients with TLB score < 0.5 (31.11% false negative rate).

**Figure 6:**
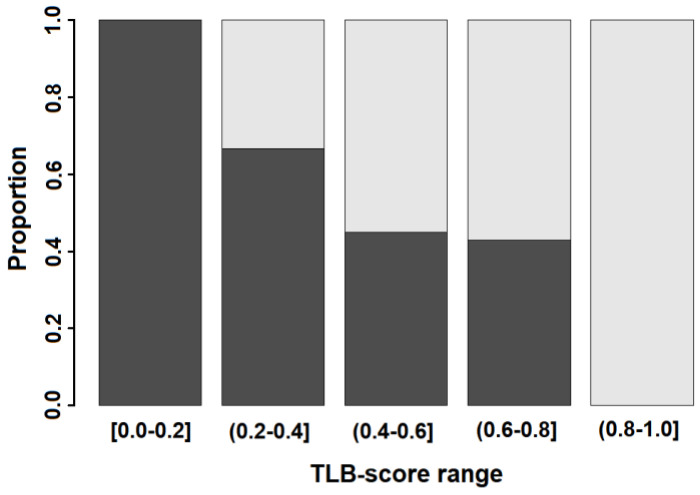
Proportion of subjects from HC subjects (dark grey) and MS patients (light grey) according to the TLB score.

Figure 5 shows the distribution of the TLB score calculated with model 3. The TLB score is a continuous variable taking values between 0 and 1; the closer to 1, the higher the probability of plasma alterations. HC and MS groups showed markedly different distribution in the TLB score: Q1 = 0.22, Q2 = 0.37 and Q3 = 0.55 for HC group, and Q1 = 0.45, Q2 = 0.64 and Q3 = 0.89 for MS group. The difference in the medians of the two groups is statistically significant (*p*-value < 0.001). This provides a visual assessment for the correlation between the TLB score and the absence or presence of MS. As the TLB score increases, the percentage of subjects predicted to be MS patients increases and the percentage of controls decreases: below a predicted TLB score of 0.25, all individuals are HC subjects, whereas above a TLB score of 0.75, all individual are patients diagnosed from MS (Figure 6).

### 3.4. Distinctive Features of MS Patients versus HC Individuals according to TLB Values

**Figure 7:**
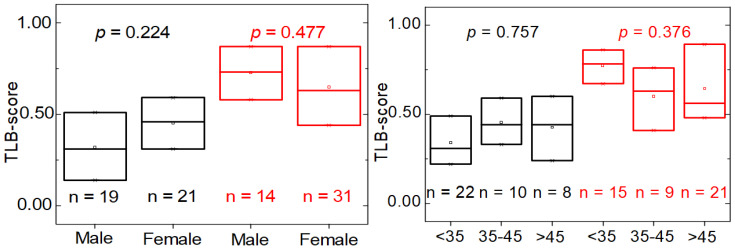
Distribution of the probability score (TLB score) within healthy individuals (HC group, black) and multiple sclerosis patients (MS group, red) according to gender (**left**) and age (**right**). The *p*-value (t-Student and ANOVA test in HC group; Wilcoxon test and Kruskal–Wallis test in MS group) indicates there is no statistically significant difference between subcategories (gender and age) within HC and MS groups (*p*-value > 0.05).

To study the performance of the proposed TLB score for detecting MS, we carried out a descriptive analysis using the data distribution in quartiles and the rank-based t-Student/Wilcoxon test or ANOVA/Kruskal–Wallis test, depending on the normality character of the parameter distribution, to address whether the distribution by group is similar or not. It can be observed (Figure 7, Table 3; Table S8) that there are no significant differences in the distribution of TLB score in the HC group according to gender and age of the individuals (*p*-value > 0.05; Table S8). Thus, in healthy individuals, the probability of having a normal/altered TLB thermogram does not depend on either gender or age. When looking at the behavior of the classification established by the TLB score, a number of subjects (13 individuals) are classified as having an altered TLB thermogram (32.50% false positives) (Table 3). There is no significant departure from independence between the variables involved (age, gender, etc.).

It is evident that the TLB score for individuals with MS is considerably higher than for HC subjects (Figure 5). As it occurred in the HC group, there are no significant differences in the TLB score according to gender or age (*p*-value > 0.05; Table 3, Table S8 and Figure 7). However, when looking at the behavior of the classification established by the TLB score, some subjects (14 patients) had an unaltered thermogram (31.11% false negatives) (Table 3). Again, in this case, there is no significant departure from independence between the variables involved.

**Figure 8:**
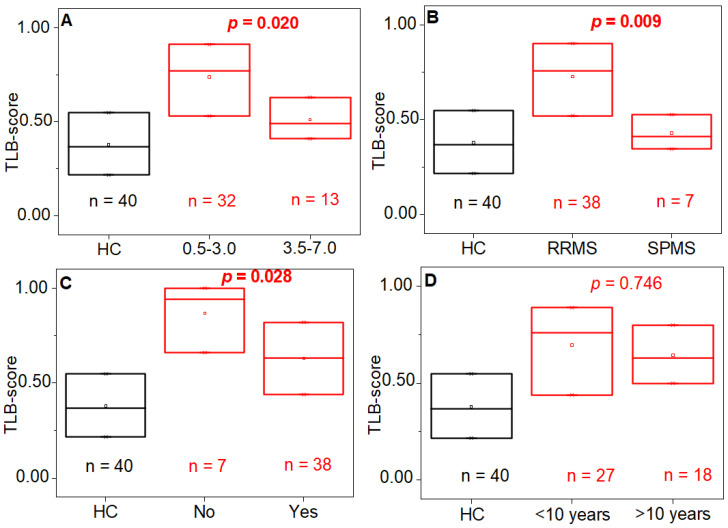
Distribution of the probability score (TLB score) for healthy individuals (HC group, black) and multiple sclerosis patients (MS group, red) according to (**A**) level of disability (EDSS = 0.5–3.0: mild disability; EDSS = 3.5–7.0: moderate/severe disability), (**B**) diagnosis stage (RRMS: early stage; SPMS: advanced stage), (**C**) pharmacological treatment (no/yes), and (**D**) disease duration (more or less than 10 years). The *p*-value (Wilcoxon test) indicates there is a statistically significant difference regarding the level of disability, the diagnosis stage and the therapy in the MS group (*p*-value < 0.05) but not in the disease duration (*p*-value > 0.05).

A statistically significant relationship was observed between the TLB score and the level of disability and diagnosis stage (*p*-value < 0.05) (Figure 8, Table S9). Moderate/severe disability (EDSS = 3.5–7.0) and advanced stage (SPMS) are characterized by small TLB scores, which would conspire to misclassify these subjects as healthy, compared to mild disability and early stage (RRMS) with higher TLB scores, which would help in identification as diseased subjects (Figure 8, Table S9). In this case, not only a significant departure from independence was observed between the variables involved, but this effect was peculiar of this analysis model and did not appear when models 1 and 2 were applied (Tables S10 and S11).

By contrast, there are no significant differences in the TLB score (model 3), in regards to the time from the onset of the disease (*p*-value = 0.746; Figure 8, Table S9).

Most of nontreated patients (n = 7), 6 out of 7 (85.71%) belonged to both EDSS (0.5–3.0) and RRMS groups. Some subjects (14 individuals) were classified as having an unaltered TLB thermogram (false negatives) and were mixed from EDSS groups (Table 4).

## 4. Discussion

A timely diagnosis of MS offers many advantages to patients, who can thus take advantage of early treatments. Attention is increasingly being focused in searching for specific biomarkers of MS in body fluids, and it would be particularly desirable to identify such markers in the bloodstream. Many efforts are ultimately directed toward the development of liquid biopsy, a powerful tool for routine clinical care in monitoring disease progression and response to therapy. In this view, recent studies aiming to explore the plasma proteome with new methodologies for sensitive detection of proteins in a wide range of concentrations have been reported [31,32]. As an example, by using a proteomic approach, a higher level of two plasma proteins (oncostatin M and hepatocyte growth factor) was found in MS patients compared to healthy subjects (correlation was proven by AUC values of 0.69 and 0.77, respectively) [32]; both proteins have a neuroprotective effect, and it was suggested that such an increase might be a natural compensation mechanism following the neuronal damage induced by the disease-associated inflammation states.

The approach proposed in our study is indirectly based on the assumption of the existence of some specific metabolites associated with blood plasma proteins and acting as biomarkers of MS, but at variance with other studies, it does not rely on the necessity of knowing their exact chemical nature, binding location, or amount. The procedure consists in taking a small amount of blood (about 3 mL) in a routine sampling and, without any preliminary special treatment or the use of any reagent, performing a simple calorimetric scan. The analysis of the plasma thermogram obtained is used to derive a single parameter called TLB score, which provides a prediction of the occurrence of MS in the subject through a comparison with the results obtained in our pilot analysis. In this work, we have compared a group of 45 MS patients with an HC group of 40 individuals. The profiles of the MS group do not show apparent differences compared to the control population, making it difficult to discriminate between the two groups by visual inspection (Figure 2). This behavior indicates that the changes within the plasma proteome of the MS patients are subtle, although we demonstrated that they are within the sensitivity of the thermal measurements. More evident differences in the thermal profiles of plasma/serum were obtained for other pathologies [25,26,28] where larger alterations of protein or metabolites may occur.

To evidence the possibility of revealing the occurrence of MS, a mathematical analysis of the calorimetric data is necessary. To this aim, we have analyzed the TLB parameters derived from the deconvolution of the experimental thermograms. Among the fourteen TLB-associated parameters extracted (Table S4), we found that three of them (*T_av_*, *AUC*_*n*4_ and *AP*_*n*4_) show statistically significant differences between healthy and diseased subjects. Based on previous studies where different algorithms were compared [27], here we selected the generalized linear model as a predictive tool that provides a TLB score which correlates with the plasma profile features. Three models were investigated (Table 2) based on the examination of data for either subgroups separately (models 1 and 2) or the complete set of the TLB-parameters (model 3). The best performance was obtained with model 3, which provided a sensitivity of 68.89% (i.e., correct classification of a diseased individual) and a specificity of 67.50% (i.e., correct classification of a healthy individual). This model, which we finally adopted for our TLB methodology, gives an area under the ROC curve value of 0.79, demonstrating a good accuracy to identify MS subjects. This finding is remarkable considering not only the similarity of the thermograms obtained for our cohort of subjects (Figure 2) but also the peculiarity of MS, which is characterized by a heterogeneous clinical presentation and disease course. The sensitivity and specificity from the model were not optimal; nevertheless, results are particularly encouraging, because (1) the visual differences between HC and MS thermograms are subtle (Figure 2); and (2) MS is a disease affecting mainly the CNS, and little is known about its reflection in blood plasma. In fact, the changes in the biochemical composition of blood plasma are relatively minor compared to changes in CSF, and they do not necessary reflect all possible alteration occurring in the CNS during the progression of the MS disease. A number of molecular biomarkers for MS disease identified in both plasma and CSF were proposed [32,33].

The distribution of the TLB score (Figure 5 and Figure 6) shows that values ≥ 0.75 are found only for MS patients, whereas TLB score ≤ 0.25 was found only for HC individuals. Within these two values, there is a partial overlap determining a 32.50% rate of false positives (HC with TLB score > 0.5) and a 31.11% rate of false negatives (MS with TLB score < 0.5). Thus, it is clear that extreme values of the TLB score are highly indicative of the presence/absence of the pathology, although we do not claim a perfect sensitivity and specificity of our methodology in such cases.

According to the results, the TLB score is not affected by gender or age in both HC and MS groups (Table 3, *p* > 0.05). When the distinctive classification features of the MS group (such as the disease stage, EDSS, and duration) are taken into account, the predictive power of the model provides better outcome for MS patients having a mild disability (EDSS ≤ 3.0, *p* = 0.020) and an early stage of the disease (RRMS, *p* = 0.009). For these patients the success rate of the TLB score is near 80% (Table 4).

These rather surprising observations seem to concur in indicating that the TLB score works best for patients with a mild form of MS and with a more recent onset. This finding mirrors the one observed when applying TLB to cancer patients [27]. A plausible explanation (and an intriguing hypothesis) is that at earlier stages of MS the metabolic disorders caused by inflammation in the CNS are considerable and they reflect in large protein-related blood plasma alterations (high TLB score), whereas when MS is at a late stage the organism has already reached a status of adaptation and compensation of the metabolic changes induced by the disease, with much less reflection in blood plasma alterations (low TLB score) [34,35,36]. Whether this hypothesis is true or not, it is interesting to note that this finding suggests a higher likelihood of success when using the TLB score for patients at an early stage of the disease, when the diagnosis is both more important and difficult.

Overall, although applied to a limited number of cases, the methodology described in this study demonstrates a good level of classification. The next steps will include determining the utility of TLB for differentiating between MS and other disorders, particularly inflammatory diseases. Further studies including more (or only) patients with both low EDSS and short onset time of the disease could give more insight on the TLB application for the decision making on MS diagnosis and follow-up in the clinical practice.

## Figures and Tables

**Table 1:** Age distribution and boundaries (Q1 to Q3) for its partition in quartiles.

Groups ^(1)^	Gender	N (%)	Minimum	Q1	Q2	Mean	Q3	Maximum
HC	Male	19 (47.50%)	24.00	26.00	32.00	36.58	40.50	71.00
	Female	21 (52.50%)	27.00	30.00	38.00	37.95	43.00	52.00
	Total	40	24.00	29.00	35.00	37.30	42.25	71.00
MS	Male	14 (31.11%)	24.00	31.25	39.00	41.21	49.50	63.00
	Female	31 (68.89%)	22.00	34.50	45.00	43.32	50.50	69.00
	Total	45	22.00	33.00	45.00	42.67	50.00	69.00
**Diagnosis ^(2)^**	**Gender**	**N (%)**	**Minimum**	**Q1**	**Q2**	**Mean**	**Q3**	**Maximum**
RRMS	Male	12 (31.58%)	24.00	31.00	33.50	38.42	47.25	58.00
	Female	26 (68.42%)	22.00	33.75	45.00	43.23	50.75	69.00
	Total	38	22.00	32.25	43.50	41.71	50.00	69.00
SPMS	Male	2 (28.57%)	53.00	55.50	58.00	58.00	60.50	63.00
	Female	5 (71.43%)	30.00	43.00	46.00	43.80	48.00	52.00
	Total	7	30.00	44.50	48.00	47.86	52.50	63.00
**EDSS ^(3)^**	**Gender**	**N (%)**	**Minimum**	**Q1**	**Q2**	**Mean**	**Q3**	**Maximum**
0.5–3.0	Male	10 (31.25%)	24.00	31.00	32.50	35.90	43.75	50.00
	Female	22 (68.75%)	22.00	33.00	42.00	41.50	50.00	69.00
	Total	32	22.00	31.00	38.00	39.75	49.25	69.00
3.5–7.0	Male	4 (30.77%)	44.00	50.75	55.50	54.50	59.25	63.00
	Female	9 (69.23%)	30.00	45.00	48.00	47.78	52.00	59.00
	Total	13	30.00	45.00	52.00	49.85	55.00	63.00
**Disease onset ^(4)^**	**Gender**	**N (%)**	**Minimum**	**Q1**	**Q2**	**Mean**	**Q3**	**Maximum**
≤10 years	Male	10 (37.04%)	29.00	31.25	33.50	39.30	47.75	58.00
	Female	17 (62.96%)	22.00	30.00	39.00	40.59	52.00	59.00
	Total	27	22.00	30.50	37.00	40.11	50.50	59.00
>10 years	Male	4 (22.22%)	24.00	39.00	48.50	46.00	55.50	63.00
	Female	14 (77.78%)	33.00	43.50	46.00	46.64	49.75	69.00
	Total	18	24.00	43.25	46.00	46.50	50.00	69.00
**Therapy ^(5)^**	**Gender**	**N (%)**	**Minimum**	**Q1**	**Q2**	**Mean**	**Q3**	**Maximum**
No	Male	2 (28.57%)	32.00	39.75	47.50	47.50	55.25	63.00
	Female	5 (71.43%)	30.00	36.00	39.00	43.80	45.00	69.00
	Total	7	30.00	34.00	39.00	44.86	54.00	69.00
Yes	Male	12 (31.58%)	24.00	31.00	39.00	40.17	48.50	58.00
	Female	26 (68.42%)	22.00	34.00	46.00	43.23	50.75	59.00
	Total	38	22.00	33.00	45.50	42.26	50.00	59.00

^(1)^: Healthy control (HC) or multiple sclerosis (MS). ^(2)^: MS patients with relapsing–remitting clinical form of MS (RRMS) and secondary progressive (SPMS) diagnosis. ^(3)^: MS patients, either with mild (expanded disability status scale (EDSS) = 0.5–3.0) or moderate/severe disability (EDSS = 3.5–7.0). ^(4)^: MS patients with short (≤ 10 years) and long (>10 years) time from the onset of the disease. ^(5)^: MS patients with (Yes) and without (No) therapy.

**Table 2:** Model comparison based on the ability to classify subjects.

Model	Success Rate	Sensitivity	Specificity	PPV	NPV
1	63.53%	64.44%	62.50%	65.91%	60.98%
2	56.47%	64.44%	47.50%	58.00%	54.29%
3	68.24%	68.89%	67.50%	70.45%	65.85%

**Table 3:** Contingency table for gender and age for model 3.

Group	Gender	TLB Score < 0.5	TLB Score > 0.5	*p*-Value
HC	Male (n = 19)	14 (73.68%)	5 (26.32%)	0.511
	Female (n = 21)	13 (61.90%)	8 (38.10%)	
MS	Male (n = 14)	2 (14.29%)	12 (85.71%)	0.165
	Female (n = 31)	12 (38.71%)	19 (61.29%)	
	**Age**	**TLB Score < 0.5**	**TLB Score > 0.5**	***p*-Value**
HC	<35 (n = 22)	17 (77.27%)	5 (22.73%)	0.315
	35–45 (n = 10)	6 (60.00%)	4 (40.00%)	
	>45 (n = 8)	4 (50.00%)	4 (50.00%)	
MS	<35 (n = 15)	3 (20.00%)	12 (80.00%)	0.444
	35–45 (n = 9)	4 (44.44%)	5 (55.56%)	
	>45 (n = 21)	7 (33.33%)	14 (66.67%)	

Note: *p*-values were calculated according to Fisher’s independence test.

**Table 4:** Contingency table for clinical history information (EDSS, diagnosis stage, disease duration and treatment) in MS group.

EDSS	TLB Score < 0.5	TLB Score > 0.5	*p*-Value
0.5–3.0 (n = 32)	7 (21.87%)	25 (78.13%)	0.072
3.5–7.0 (n = 13)	7 (53.85%)	6 (46.15%)	
**Diagnosis**	**TLB Score < 0.5**	**TLB Score > 0.5**	***p*-Value**
RRMS (n = 38)	9 (23.68%)	29 (76.32%)	0.023
SPMS (n = 7)	5 (71.43%)	2 (28.57%)	
**Disease duration**	**TLB Score < 0.5**	**TLB Score > 0.5**	***p*-Value**
<10 years (n = 27)	9 (33.33%)	18 (66.67%)	0.753
>10 years (n = 18)	5 (27.78%)	13 (72.22%)	
**Therapy**	**TLB Score < 0.5**	**TLB Score > 0.5**	***p*-Value**
No (n = 7)	0 (0.00%)	7 (100.00%)	0.081
Yes (n = 38)	14 (36.84%)	24 (63.16%)	

Note: *p*-values were calculated according to Fisher’s independence test.

## Data Availability

The data presented in this study are available from the corresponding authors upon reasonable request.

## Supplementary Materials

The following are available online at https://www.mdpi.com/article/10.3390/jpm11040295/s1, Table S1: monovariant analysis of TLB parameters of age groups; Table S2: monovariant analysis of TLB parameters of MS group according to EDSS; Table S3: main descriptive indexes for the fourteen TLB-associated parameters; Table S4: monovariant analysis of TLB parameters of HC and MS groups; Table S5: ROC analysis for individual TLB-associated parameters; Table S6: Summary of the application of binomial generalized linear model with logistic regression (GLM) to the three models; Table S7: model comparison based on the likelihood ratio test; Table S8: TLB score (model 3) vs. gender and age; Table S9: TLB score (model 3) vs. clinical history information (EDSS, diagnosis stage, disease duration and therapy) for MS group. Table S10: Contingency table for clinical history information (EDSS, diagnosis stage, disease duration, and therapy) for MS group (using TLB score from model 1); Table S11: contingency table for clinical history information (EDSS, diagnosis stage, disease duration, and therapy) for MS group (using TLB score from model 2).
