# Supplementary material for: Thermal Liquid Biopsy (TLB) of Blood Plasma as a Potential Tool to Help in the Early Diagnosis of Multiple Sclerosis
Source: J Pers Med. 2021 Apr 13;11(4):295. doi: 10.3390/jpm11040295 (PMC8069382; doi:10.3390/jpm11040295)
Supplement: Supplementary file 1 [file jpm-11-00295-s001.pdf]

Supplementary Information

Thermal Liquid Biopsy (TLB) of Blood Plasma as a Potential Tool to Help in the Early Diagnosis of Multiple Sclerosis

Ferdinanda Annesi, Sonia Hermoso-Durán, Bruno Rizzuti, Rosalinda Bruno, Domenico Pirritano, Alfredo Petrone, Francesco Del Giudice, Jorge Ojeda, Sonia Vega, Oscar Sanchez-Gracia, Adrian Velazquez-Campoy, Olga Abian and Rita Guzzi

Table S1. *Monovariant analysis of TLB parameters of age groups.*

Parameter	<35.00 (n=37)	35.00-45.00 (n=19)	>45.00 (n=29)	p-value
T_{av}	68.79 (0.62)	68.68 (0.77)	68.59 (0.77)	0.548
G_1	0.22 (0.14)	0.15 (0.12)	0.22 (0.14)	0.191
AUC_{n2}	36.32 [24.12;144.45]	40.09 [30.91;84.43]	50.93 [31.83;80.14]	0.963
AP_{n2}	1.80 [0.58;30.79]	2.42 [1.29;12.17]	3.99 [1.11;9.43]	0.975
AUC_{n3}	22.80 [18.13;51.87]	25.98 [18.65;35.54]	21.30 [19.13;26.42]	0.838
AP_{n3}	0.76 [0.47;2.83]	0.82 [0.53;1.25]	0.72 [0.54;0.81]	0.890
AUC_{n4}	30.30 [25.62;38.37]	30.73 [28.04;35.65]	31.70 [28.01;40.84]	0.634
AP_{n4}	2.56 [2.12;3.72]	2.55 [2.01;3.26]	2.83 [2.03;4.19]	0.885
AUC_{n5}	45.39 [41.06;52.27]	44.58 [39.09;51.17]	46.17 [40.34;50.39]	0.699
AP_{n5}	2.56 [2.12;3.72]	2.55 [2.01;3.26]	2.83 [2.03;4.19]	0.885
Dv_2	1.11 [1.00;1.31]	1.03 [0.95;1.22]	1.06 [0.99;1.14]	0.406
Dv_3	1.03 [0.95;1.24]	0.98 [0.95;1.15]	1.08 [0.99;1.25]	0.358
Dv_4	0.72 [0.47;1.09]	0.58 [0.31;0.93]	0.79 [0.54;1.17]	0.373
Dv_5	0.65 [0.53;0.96]	0.51 [0.25;0.97]	0.65 [0.45;1.05]	0.389

Note: *average (standard deviation) and median [Q1;Q3] are provided depending on the normality character of the parameter distribution.*

Table S2. *Monovariant analysis of TLB parameters of MS group according to EDSS.*

Parameter	EDSS=0.50-3.00 (n=32)	EDSS=3.50-7.00 (n=13)	p-value
T_{av}	68.48 [68.14;68.93]	68.89 [68.69;69.24]	**0.011**
G_1	0.19 (0.12)	0.22 (0.17)	0.482
AUC_{n2}	37.82 [26.51;83.50]	38.43 [31.96;54.25]	0.900
AP_{n2}	1.74 [0.73;9.96]	2.33 [1.35;5.34]	0.783
AUC_{n3}	25.19 [21.08;35.56]	23.51 [20.01;29.48]	0.634
AP_{n3}	0.76 [0.56;1.20]	0.80 [0.63;1.01]	0.900
AUC_{n4}	33.46 [28.13;43.78]	31.06 [30.17;37.24]	0.689
AP_{n4}	3.19 [1.99;4.23]	2.39 [1.71;3.37]	0.381
AUC_{n5}	47.53 [41.46;57.26]	42.15 [40.34;49.40]	0.139
AP_{n5}	3.19 [1.99;4.23]	2.39 [1.71;3.37]	0.381
Dv_2	1.06 [0.99;1.14]	1.11 [0.99;1.15]	0.726
Dv_3	1.04 [0.94;1.10]	1.09 [0.94;1.25]	0.367
Dv_4	0.76 [0.53;1.42]	0.80 [0.41;1.20]	0.652
Dv_5	0.62 [0.47;1.01]	0.69 [0.47;0.87]	0.802

Note: *Average (standard deviation) and median [Q1;Q3] are provided depending on the normality character of the parameter distribution.*

Monoparametric descriptive analysis

A monovariant analysis with each single TLB-associated parameter was performed to determine statistically significant differences between HC and MS groups. Previously, a normality test was performed for each parameter, and, therefore, the t-Student test (normal parameter) or the Wilcoxon test (non-normal parameter) was applied.

Table S3. Main descriptive indexes for the fourteen TLB-associated parameters.

HC	Minimum	Q1	Q2	Average	Q3	Maximum
T_{av}	67.64	68.45	68.94	68.89	69.25	70.35
G_1	-0.09	0.12	0.21	0.21	0.32	0.44
AUC_{n2}	16.66	25.56	66.07	135.15	159.30	927.47
AP_{n2}	0.10	0.84	6.06	70.79	38.46	1237.47
AUC_{n3}	14.46	17.72	20.67	73.97	46.09	444.99
AP_{n3}	0.36	0.48	0.61	11.92	2.08	138.76
AUC_{n4}	19.02	25.35	27.82	29.99	34.32	46.26
AP_{n4}	0.20	0.83	1.10	1.21	1.58	3.26
AUC_{n5}	34.18	39.90	42.42	49.10	52.32	128.17
AP_{n5}	0.81	2.12	2.53	3.20	3.42	14.14
Dv_2	0.73	0.96	1.10	1.58	1.31	16.48
Dv_3	0.36	0.98	1.07	1.73	1.36	10.65
Dv_4	0.16	0.47	0.71	0.73	0.95	1.869
Dv_5	0.05	0.39	0.64	0.77	0.97	3.42
**MS**	**Minimum**	**Q1**	**Q2**	**Average**	**Q3**	**Maximum**
T_{av}	66.31	68.23	68.61	68.52	68.94	69.75
G_1	-0.11	0.10	0.19	0.20	0.31	0.53
AUC_{n2}	17.44	27.25	38.29	87.77	70.90	666.80
AP_{n2}	0.22	0.77	1.80	22.25	6.82	279.56
AUC_{n3}	15.41	20.99	24.64	32.44	30.25	134.95
AP_{n3}	0.35	0.56	0.77	1.45	1.08	13.15

AUC_{n4}	19.72	28.33	33.31	42.86	42.66	181.42
AP_{n4}	0.42	1.09	1.42	2.72	2.27	19.69
AUC_{n5}	26.79	40.86	47.09	52.57	51.35	175.20
AP_{n5}	0.43	1.87	2.87	3.90	4.19	33.43
Dv_2	0.44	0.99	1.06	1.16	1.14	2.95
Dv_3	0.30	0.94	1.04	1.07	1.11	1.79
Dv_4	0.06	0.49	0.79	1.73	1.38	15.22
Dv_5	0.13	0.47	0.65	0.94	0.99	9.45

Table S4. *Monovariant analysis of TLB parameters of HC and MS groups.*

**Parameter**	**HC (n=40)**	**MS (n=45)**	**p-value**
T_{av}	68.89 (0.62)	68.52 (0.74)	**0.013**
G_1	0.21 (0.14)	0.20 (0.14)	0.555
AUC_{n2}	66.07 [25.56;159.30]	38.30 [27.26;70.90]	0.311
AP_{n2}	6.06 [0.84;38.46]	1.80 [0.77;6.82]	0.249
AUC_{n3}	20.67 [17.73;46.09]	24.64 [20.99;30.25]	0.252
AP_{n3}	0.62 [0.48;2.08]	0.77 [0.56;1.08]	0.520
AUC_{n4}	27.82 [25.35;34.32]	33.31 [28.33;42.66]	**0.002**
AP_{n4}	1.11 [0.83;1.58]	1.42 [1.09;2.27]	**0.010**
AUC_{n5}	42.42 [39.90;52.32]	47.09 [40.86;51.35]	0.423
AP_{n5}	2.53 [2.12;3.42]	2.87 [1.87;4.19]	0.549
Dv_2	1.10 [0.96;1.31]	1.06 [0.99;1.14]	0.438
Dv_3	1.07 [0.98;1.36]	1.04 [0.94;1.11]	0.098
Dv_4	0.71 [0.47;0.95]	0.79 [0.49;1.38]	0.208
Dv_5	0.64 [0.39;0.97]	0.65 [0.47;1.00]	0.888

Note: *Average (standard deviation) and median [Q1;Q3] are provided depending on the normality character of the parameter distribution.*

Table S5. *ROC analysis for individual TLB-associated parameters.*

Parameter	Success Rate	Sensitivity	Specificity	Threshold	Trend
T_{av}	37.65%	26.67%	50.00%	68.94	↓
G_1	44.71%	42.22%	47.50%	0.20	↑
AUC_{n2}	36.47%	26.67%	47.50%	64.45	↑
AP_{n2}	36.47%	17.78%	57.50%	15.92	↑
AUC_{n3}	64.71%	75.56%	52.50%	20.90	↑
AP_{n3}	62.35%	80.00%	42.50%	0.53	↑
AUC_{n4}	67.06%	71.11%	62.50%	30.05	↑
AP_{n4}	64.71%	80.00%	47.50%	1.06	↑
AUC_{n5}	61.18%	55.55%	67.50%	46.15	↑
AP_{n5}	58.82%	46.67%	72.50%	3.20	↓
Dv_2	38.82%	22.22%	57.50%	1.16	↑
Dv_3	41.18%	68.89%	10.00%	0.95	↑
Dv_4	50.59%	93.33%	2.50%	0.17	↑
Dv_5	55.29%	77.78%	30.00%	0.44	↑

Note: *The Youden method was employed to calculate the threshold, for each individual parameter, for classifying subjects as healthy (negative result, TLB score < 0.5, unaltered TLB thermogram) and diseased (positive result, TLB score > 0.5, altered TLB thermogram). The trend symbol indicates the direction for classification as a healthy subject: ↑↓ means that, in general, subjects classified as healthy (negative result according to TLB) showed values that were larger/smaller than the indicated threshold. The success rate or accuracy is the probability of correctly classifying subjects as healthy (unaltered TLB thermogram and belonging to the HC group) or diseased (altered TLB thermogram and belonging to the MS group).*

Multiparametric predictive/classifying TLB score for Multiple Sclerosis based on GLM

Although some of the individual TLB-associated parameters exhibit good performance regarding subject classification in HC and MS groups, the combined use of several parameters (provided that the two single-parameter preliminary approaches have provided slightly different subsets of relevant parameters) improves the performance of the classification score with the goal of applying TLB in clinical practice. Three models, each using a different set of parameters, were considered. Model 1 was constructed based on T_{av}, G_1, AUC_{ni}, and AP_{ni}; Model 2 was constructed based on Dv_i; and Model 3 considered all 14 parameters.

TLB scores were elaborated applying GLM with the three Models 1-3. Because the Dv_i parameters are non-linear functions of other individual TLB-associated parameters, establishing whether or not Model 3 represents a significant improvement (compared to Model 1) for predicting a health/disease condition was a required step. After applying GLM to each set of parameters, none of the parameters shows statistical significance (all p-values > 0.05) for discriminating between HC and MS groups (*Table S4*).

Table S6*. Summary of the application of Binomial Generalized Linear Model with Logistic Regression (GLM) to the three models.*

	**Parameter**	**z value**	***p*-value**
Model 1	T_{av}	-1.08	0.279
	G_1	-0.34	0.734
	AUC_{n2}	-0.13	0.899
	AP_{n2}	-0.21	0.837
	AUC_{n3}	0.53	0.593
	AP_{n3}	-0.89	0.375
	AUC_{n4}	1.46	0.144
	AP_{n4}	-0.79	0.427
	AUC_{n5}	1.16	0.247
	AP_{n5}	-0.69	0.488
Model 2	Dv_2	-0.71	0.478
	Dv_3	-1.49	0.135
	Dv_4	1.61	0.106

	Dv_5	0.71	0.479
Model 3	T_{av}	-0.77	0.444
	G_1	-0.32	0.752
	AUC_{n2}	0.92	0.358
	AP_{n2}	-1.01	0.310
	AUC_{n3}	0.28	0.778
	AP_{n3}	-0.34	0.731
	AUC_{n4}	1.34	0.179
	AP_{n4}	-0.69	0.487
	AUC_{n5}	-0.11	0.913
	AP_{n5}	0.56	0.574
	Dv_2	0.99	0.320
	Dv_3	-0.15	0.882
	Dv_4	0.37	0.713
	Dv_5	-0.78	0.433

Note: *A cut-off value of 2.00 for z-value (which approximately corresponds to a two-sided hypothesis test with a significance level of α = 0.05) indicates whether the corresponding parameters are statistically meaningful in the model. In other words, if the absolute value of the z-value is larger than 2.00, then its corresponding GLM coefficient is non-negligible, i.e., not null, and the parameter is important and meaningful for the prediction of the presence/absence of MS.*

The model equivalence test, based on the likelihood ratio, shows that Model 1 and 2 can be considered statistically equivalent to Model 3 (*Table S5*), but Model 3 shows smaller residual deviance.

Table S7. *Model comparison based on the likelihood ratio test.*

	Model 1	Model 2	Model 3
Degrees of Freedom	-4.00	-10.00	n.a.
Residual Degrees of Freedom	74.00	80.00	70.00
Residual Deviance	89.26	100.38	87.89
Equivalency with Model 3 $P(\chi^2)$	0.850	0.253	n.a.

n.a.: Not applicable.

Table S8. *TLB score (Model 3) vs. gender and age.*

	HC					MS				
**Gender**	**N**	**Q1**	**Q2**	**Q3**	**p-value**	**N**	**Q1**	**Q2**	**Q3**	**p-value**
Male	19	0.14	0.31	0.51	0.224	14	0.58	0.73	0.87	0.477
Female	21	0.31	0.46	0.59		31	0.44	0.63	0.87	
**Age**	**N**	**Q1**	**Q2**	**Q3**	**p-value**	**N**	**Q1**	**Q2**	**Q3**	**p-value**
<35	22	0.22	0.31	0.49		15	0.67	0.78	0.86	
35-45	10	0.33	0.44	0.59	0.757	9	0.41	0.63	0.76	0.376
>45	8	0.24	0.44	0.60		21	0.48	0.56	0.89	

Note: *p-values were calculated according to t-Student and ANOVA test in HC group; Wilcoxon test and Kruskal-Wallis test in MS group.*

Table S9. *TLB score (Model 3) vs. clinical history information (EDSS, diagnosis stage, disease duration and therapy) for MS group.*

**EDSS**	**N**	**Q1**	**Q2**	**Q3**	**p-value**
0.5-3.0	32	0.53	0.77	0.91	**0.020**
3.5-7.0	13	0.41	0.49	0.63	
**Diagnosis Stage**	**N**	**Q1**	**Q2**	**Q3**	**p-value**
RRMS	38	0.52	0.76	0.90	**0.009**
SPMS	7	0.34	0.41	0.53	
**Disease duration**	**N**	**Q1**	**Q2**	**Q3**	**p-value**
<10 years	27	0.44	0.76	0.89	0.746
>10 years	18	0.50	0.63	0.80	
**Therapy**	**N**	**Q1**	**Q2**	**Q3**	**p-value**
No	7	0.66	0.94	1.00	**0.028**
Yes	38	0.44	0.63	0.82	

Note: *p-values were calculated according to Wilcoxon test.*

Table S10. *Contingency table for clinical history information (EDSS, diagnosis stage, disease duration and therapy) for MS group (using TLB score from Model 1).*

**Group**	**EDSS**	**TLB score < 0.5**	**TLB score > 0.5**	**p-value**
MS	0.5-3.0 (n=32)	9 (28.12%)	23 (71.88%)	0.169
	3.5-7.0 (n=13)	7 (53.85%)	6 (46.15%)	
	**Diagnosis**	**TLB score < 0.5**	**TLB score > 0.5**	**p-value**
MS	RRMS (n=38)	11 (28.95%)	27 (71.05%)	0.079
	SPMS (n=7)	5 (71.43%)	2 (28.57%)	
	**Disease duration**	**TLB score < 0.5**	**TLB score > 0.5**	**p-value**
MS	<10 years (n=27)	9 (33.33%)	18 (66.67%)	0.758
	>10 years (n=18)	7 (38.89%)	11 (61.11%)	
	**Therapy**	**TLB score < 0.5**	**TLB score > 0.5**	**p-value**
MS	No (n=7)	1 (14.29%)	6 (85.71%)	0.393
	Yes (n=38)	15 (39.47%)	23 (60.53%)	

Note: *p-values were calculated according to Fisher's independence test.*

Table S11. *Contingency table for clinical history information (EDSS, diagnosis stage, disease duration & therapy) for MS group (using TLB score from Model 2).*

Group	EDSS	TLB score < 0.5	TLB score > 0.5	p-value
MS	0.5-3.0 (n=32)	9 (28.12%)	23 (71.88%)	0.169
	3.5-7.0 (n=13)	7 (53.85%)	6 (46.15%)	
	**Diagnosis**	**TLB score < 0.5**	**TLB score > 0.5**	**p-value**
MS	RRMS (n=38)	13 (34.21%)	25 (65.79%)	0.686
	SPMS (n=7)	3 (42.86%)	4 (57.14%)	
	**Disease duration**	**TLB score < 0.5**	**TLB score > 0.5**	**p-value**
MS	<10 years (n=27)	8 (29.63%)	19 (70.37%)	0.354
	>10 years (n=18)	8 (44.44%)	10 (55.56%)	
	**Therapy**	**TLB score < 0.5**	**TLB score > 0.5**	**p-value**
MS	No (n=7)	1 (14.29%)	6 (85.71%)	0.393
	Yes (n=38)	15 (39.47%)	23 (60.53%)	

Note: *p-values were calculated according to Fisher's independence test.*
